# Molecular Mechanisms Underpinning Aggregation in *Acidiphilium* sp. C61 Isolated from Iron-Rich Pelagic Aggregates

**DOI:** 10.3390/microorganisms8030314

**Published:** 2020-02-25

**Authors:** Qianqian Li, Rebecca E. Cooper, Carl-Eric Wegner, Kirsten Küsel

**Affiliations:** 1 Institute of Biodiversity, Friedrich Schiller University Jena, 07743 Jena, Germany; qianqian.li@uni-jena.de (Q.L.); rebecca.cooper@uni-jena.de (R.E.C.); carl-eric.wegner@uni-jena.de (C.-E.W.); 2 The German Centre for Integrative Biodiversity Research (iDiv) Halle-Jena-Leipzig, 04103 Leipzig, Germany

**Keywords:** 2-phenethylamine (PEA), microbial aggregation, iron snow, genomics, pangenomics, RNA-seq

## Abstract

Iron-rich pelagic aggregates (iron snow) are hot spots for microbial interactions. Using iron snow isolates, we previously demonstrated that the iron-oxidizer *Acidithrix* sp. C25 triggers *Acidiphilium* sp. C61 aggregation by producing the infochemical 2-phenethylamine (PEA). Here, we showed slightly enhanced aggregate formation in the presence of PEA on different *Acidiphilium* spp. but not other iron-snow microorganisms, including *Acidocella* sp. C78 and *Ferrovum* sp. PN-J47. Next, we sequenced the *Acidiphilium* sp. C61 genome to reconstruct its metabolic potential. Pangenome analyses of *Acidiphilium* spp. genomes revealed the core genome contained 65 gene clusters associated with aggregation, including autoaggregation, motility, and biofilm formation. Screening the *Acidiphilium* sp. C61 genome revealed the presence of autotransporter, flagellar, and extracellular polymeric substances (EPS) production genes. RNA-seq analyses of *Acidiphilium* sp. C61 incubations (+/− 10 µM PEA) indicated genes involved in energy production, respiration, and genetic processing were the most upregulated differentially expressed genes in the presence of PEA. Additionally, genes involved in flagellar basal body synthesis were highly upregulated, whereas the expression pattern of biofilm formation-related genes was inconclusive. Our data shows aggregation is a common trait among *Acidiphilium* spp. and PEA stimulates the central cellular metabolism, potentially advantageous in aggregates rapidly falling through the water column.

## 1. Introduction

Pelagic aggregates, composed of microorganisms, phytoplankton, feces, detritus, and biominerals, are local hotspots for microbial interaction in nearly all aquatic habitats [1,2,3]. These snow-like aggregates are stabilized by a matrix of extracellular polymeric substances (EPS) and vary in size, ranging from micrometers to centimeters, depending on their residence time in the water column and the trophic state of the ecosystem [1,4,5]. Microbial colonization and coordinated group behavior within these pelagic aggregates are likely regulated by chemical signaling, including quorum sensing signaling molecules [6,7]; however, most chemical mediators involved in interspecies interaction are still unknown.

Iron-rich pelagic aggregates (iron snow), analogous to the more organic-rich marine or freshwater aggregates, are characterized by lower chemical and microbial complexity [5]. Iron snow forms at the redoxcline of stratified iron-rich lakes, where the oxygen-rich epilimnion water meets ferrous iron (Fe^2+^) of the anoxic hypolimnion [5]. Many of these lakes are acidic due to the inflow of protons in addition to Fe^2+^ and sulfate (SO_4_^2-^) from mine tailings [8,9]. Under acidic conditions, Fe^2+^ is oxidized by microorganisms to ferric iron (Fe^3+^), from which goethite and schwertmannite form via hydrolysis of Fe^3+^ cations [10,11]. In lignite mine lakes, these biominerals form the main inorganic component of pelagic aggregates [12,13]. These aggregates are stabilized by the adsorption of other metals, nutrients, and organic matter. Iron snow is an attractive habitat for heterotrophic microorganisms, especially those capable of using Fe^3+^ as an electron acceptor, such as *Acidiphilium* species [14]. Together, iron-oxidizing bacteria (FeOB) and iron-reducing bacteria (FeRB) can comprise up to 60% of the total microbial community found in iron snow aggregates [15]. To study the interactions between these iron-cycling bacteria, we isolated several key players from iron snow, including *Acidiphilium*, *Acidocella*, *Acidithrix,* and *Ferrovum* species [13,16,17]. The iron-oxidizing isolate *Acidithrix* sp. C25 forms large cell-mineral aggregates in the late stationary phase [13]. When co-cultured with the iron-reducing isolate *Acidiphilium* sp. C61, motile cells of *Acidiphilium* also form cell aggregates with similar morphology to iron snow. Comparative metabolomics identified the aggregation-inducing signal, 2-phenethylamine (PEA), which also induced faster growth of *Acidiphilium* sp. C61 [17].

PEA is a small molecule that exhibits an array of seemingly unrelated functions, including roles as a neurotransmitter and in food processing [18]. PEA was found in the brains of humans and other mammals [19] and reportedly has stimulatory effects, resulting in the release of biogenic amines [20]. In high concentrations, PEA can act as an anti-microbial against *Escherichia coli* on beef meat [18]. Bacteria can produce PEA via decarboxylation of phenylalanine or as a by-product of the tyrosine decarboxylase reaction [21]. PEA is capable of inhibiting both swarming and the expression of the *flhDC* gene cluster, which encodes a flagellar regulon that regulates flagellar motility in *Proteus mirabilis* [22,23]. Swarmer cell differentiation is dependent on specific environmental conditions, including the presence of a solid surface, inhibition of flagellar rotation, and density-based cell–cell signaling by extracellular signals [24,25,26]. However, swarming is not known to exist in *Acidiphilium* spp. and this *flhDC* gene cluster is absent in all sequenced *Acidiphilium* spp. genomes [17]. Therefore, the molecular mechanisms underlying PEA-induced aggregate formation in *Acidiphilum* spp. remain unknown.

To broaden our understanding of chemical communication between iron-cycling bacteria shaping pelagic aggregates, we amended different *Acidiphilium* spp. and two other iron snow key players with PEA to see if this aggregation effect was isolate specific. We sequenced the genome of *Acidphilium* sp. C61 to gain more insights into the metabolic pathways and potential behaviors (e.g., motility, chemotaxis) of this organism. Furthermore, we performed comparative transcriptomics of *Acidphilium* sp. C61 amended with 10 μM PEA compared to cultures without PEA to elucidate the genetic mechanisms underlying aggregate formation.

## 2. Materials and Methods

### 2.1. Bacterial Strains, Growth Conditions, and Microscopic Characterization of Aggregate Formation in Acidophilic Bacteria

For incubation studies, three different Fe-reducing *Acidiphilium* spp. (*Acidiphilium* sp. C61, *Acidiphilium cryptum* JF-5, and *Acidiphilium* SJH) isolated from different environments were used. Briefly, *Acidiphilium* sp. C61 was isolated just below the redox cline in the water column of the central basin (pH 2.8–3.0) of lignite mine Lake 77 (Lusatian mining area in east-central Germany) [13,17], *A. cryptum* JF-5, isolated from Lake 77 sediments [14], and *Acidiphilium* SJH (strain kindly provided by Barrie Johnson, School of Natural Sciences, Bangor University) was originally isolated from an abandoned pyrite mine in North Wales [27]. In addition, we tested the FeRB *Acidocella* sp. C78, isolated from the Lake 77 water column [17], and the FeOB *Ferrovum* sp. PN-J47 (strain kindly provided by Michael Schlömann, Technical University Bergakademie Freiberg) [28] to determine the effect of two different concentrations of 2-Phenethylamine (PEA) (Alfa Aesar, Kandel, Germany) (10 and 50 µM) on potential aggregate formation in monoculture incubations. Incubations were carried out using a defined medium, artificial pilot-plant water (APPW) medium (pH 2.5), and prepared as previously described (0.022 g L^−1^ K_2_SO_4_, 3.24 g L^−1^ MgSO_4_·7H_2_O, 0.515 g L^−1^ CaSO_4_·2H_2_O, 0.058 g L^−1^ NaHCO_3_, 0.010 g L^−1^ NH_4_Cl, 0.014 g L^−1^ Al_2_(SO_4_)_3_·18H_2_O, 0.023 g L^−1^ MnCl_2_·4H_2_O, 0.0004 g L^−1^ ZnCl_2_) [28] with the exception of added yeast extract (0.2 g L^−1^) to ensure growth of *Acidiphilium* and *Acidocella* strains [14,17]. All incubations were grown aerobically at room temperature on a rotary shaker (100 rpm) for three days.

All incubations were prepared in triplicate and growth was monitored spectrophotometrically (OD_600nm_) using a DR3900 spectrometer (Hach Lange, Düsseldorf, Germany) or indirectly using the 1,10-phenanthroline method [29] to monitor the oxidation of Fe^2+^ (*Ferrovum* sp. PN-J47). *Acidiphilium* spp., *Acidocella* sp. C78, and *Ferrovum* sp. PN-J47 were grown to exponential phase (OD_600nm_ = 0.03; A_512nm_ = 1.5) in the presence of 0, 10, or 50 µM PEA. Then, 10 µM SYTO 13 (Thermo Scientific, Schwerte, Germany) was used to stain nucleic acids of cells in all subsamples taken. Additionally, 8 μL of each cell culture was stained with SYTO 13 and placed on glass microscope slides. Aggregate formation was visually examined using an Axioplan fluorescence microscope (Zeiss, Oberkochen, Germany).

An additional 2 mM glucose was added to the above *Acidiphilium* sp. C61 culture supplemented with 0, 10, 50 µM PEA. Sterile pre-processed glass slides (Roth, Karlsruhe, Germany), which were immersed in H_2_O_2_: HNO_3_ (1:1 *v/v*, Roth, Karlsruhe, Germany) overnight, were submerged in 50 mL conical tubes with 35 mL cultures. Glass slides were taken out and applied 50 μL SYTO 13 to stain nucleic acids of cells in the biofilm. Biofilm formation was visually examined using the above Axioplan fluorescence microscope.

### 2.2. Genomic DNA Extraction and Whole Genome Sequencing of Acidiphilium sp. C61

*Acidiphilium* sp. C61 genomic DNA was extracted using a phenol–chloroform based method from cultures grown to an OD_600nm_ of 0.060 in APPW+YE medium. Briefly, biomass was harvested by centrifugation at 12,000× *g* for 10 min at room temperature and subjected to bead beating (6.5 m s^−1^ for 30 s with 0.6 g Zirconium/glass-Beadsbeads (⌀ = 1 mm. Carl Roth, Karlsruhe, Germany) in 750 µL sodium phosphate buffer (120 mM, pH 8.0) plus 250 µL TNS solution (500 mM Tris-HCl pH 8.0, 100 mM NaCl, 10% SDS *w/v*). Cell debris was separated by centrifugation. Nucleic acid extraction of the supernatant was performed in two sequential steps: first with phenol–chloroform–isoamyl alcohol (25:24:1 *v/v/v*, AppliChem, Darmstadt, Germany) and second with chloroform–isoamyl alcohol (24:1 *v/v*, AppliChem, Darmstadt, Germany). The aqueous phase was precipitated overnight at −20 °C with two volumes of polyethylene glycol 6000 (Carl Roth, Karlsruhe, Germany). Glycogen (20 mg mL^−1^, Sigma-Aldrich, Darmstadt, Germany) was added to facilitate precipitation. DNA was collected by centrifugation (20,000× *g* at 4 °C for 90 min). The resulting pellets were washed with ice-cold ethanol (70% *v/v*), centrifuged, and resuspended in 50 µL elution buffer (Qiagen, Hilden, Germany). Genomic DNA was sent to RTL genomics (Lubbock, TX, USA) for library preparation and whole genome sequencing using PacBio^®^ SMRTBell reagents (Pacific Biosciences, Menlo Park, CA, USA) and a PacBio RSII instrument.

### 2.3. Genome Assembly and Annotation

RTL genomics performed initial quality control and assembly of the *Acidiphilium* sp. C61 whole genome sequence. Raw data were subjected to quality control using FastQC (v. 0.11.7) (http://www.bioinformatics.babraham.ac.uk/projects/fastqc/) and assembled using HGAP3 (Hierarchical Genome Assembly Process 3 ) implemented in the SMRTLink software suite (v. 4.0) [30]. Basic genome characteristics were determined with QUAST (v. 4.0) [31]. Genome completeness and contamination level were estimated using the lineage-specific workflow of CheckM (v. 1.0.12) [32] with default settings, except for the “reduced_tree” parameter, which was applied to reduce the computational demand. The contamination percentage provided by CheckM represents the redundancy of single copy marker genes in this *Acidiphilium* sp. C61 genome sequence. The assembled genome was subsequently annotated using dfast (v. 1.1.0) [33] with default settings. This annotation was complemented by additional BlastKOALA searches [34] with default settings of encoded amino acid sequences against the KEGG GENES database. We collected amino acid sequences of known autotransporters and iron reductases from the NCBI non-redundant protein database by text searches. These sequences were clustered based on a sequence identity of 90% using CD-HIT (v. 4.7) [35] and used as queries for diamond (v. 0.9.26.127) searches with default settings [36] against the genome of *Acidiphilium* sp. C61 to identify potential genes encoding known autotransporters and iron reductases.

### 2.4. Pangenomic Analysis

We collected six publically available genomes from the GenBank Assembly Database of related *Acidiphilium* spp., including *A. cryptum* JF-5 (GenBank assembly accession: GCA_000016725.1), *A. multivorum* AIU301 (GCA_000202835.1), *A. angustum* ATCC 35903 (GenBank assembly accession: GCA_000701585.1), *A. rubrum* ATCC 35905 (GCA_900156265.1), *Acidiphilium* sp. JA12-A1 (GCA_000724705.2), and *Acidiphilium* sp. PM (GCA_000219295.2). We performed a pangenomic analysis combining the six publically available *Acidiphilium* spp. genomes and our *Acidiphilium* sp. C61 isolate genome using anvi’o (v. 5.5), following a previously published workflow [37,38]. Briefly, the headers of the retrieved genome fasta files were simplified using the program “anvi-script-reformat-fasta”. The “anvi-gen-contigs-database”, which incorporates prodigal (v. 2.6.3) to identify open reading frames (ORFs), was used to profile *Acidiphilium* spp. genomes [39]. Genes were annotated with the program “anvi-run-ncbi-cogs” based on blastp (v. 2.5.0) [40], which searches against the December 2014 release of the Clusters of Orthologous Groups (COGs) database [41]. In this study, we define a pangenome as the whole gene set of all strains of *Acidiphilium* sp. We use the term “core genes” to describe all genes present in all *Acidiphilium* genomes and the term “accessory genes” when we discuss genes present in a single or multiple *Acidiphilium* genomes. The *Acidiphilium* pangenome was computed using the program “anvi-pan-genome” (settings: minbit: 0.5, mcl-inflation: 10), which relies on blastp for calculating amino acid sequence similarities across genomes, the minbit heuristic first implemented in ITEP [42] to identify and remove weak amino acid sequence matches, and the MCL algorithm [43] to identify clusters based on amino acid sequence similarity searches. The results were displayed using the program “anvi-display-pan” and analyzed using the interactive interface of anvi’o.

### 2.5. 16S rRNA Gene Phylogenetic Analysis

16S rRNA gene sequences from *Acidiphilium* sp. C61 (LN866588.1), *A. cryptum* JF-5 (Y18446.1), *A. multivorum* AIU301 (NR_074327.1), *A.* SJH (AY040740.1), *A. rubrum* (D30776.1), *A. angustum* (D30772.1), *Acidocella* sp. C78 (LN866590.1), *Acidocella aminolytica* (D30771.1), and *Acidocella facilis* (D30774.1), *Acidisphaera rubrifaciens* (NR_037119.1), *Acidicaldus organivorans* strain Y008 (NR_042752.1) from Acetobacteraceae, *Magnetospirillum gryphiswaldense* MSR-1 (NR_121771.1), *Magnetospirillum marisnigri* strain SP-1 (NR_149242.1), *Magnetospirillum magnetotacticum* strain DSM 3856 (NR_026381.1), *Magnetospirillum caucaseum* strain SO-1 (NR_149241.1) from Rhodospirillaceae, *Acidobacterium capsulatum* (D26171.1), *Acidobacterium ailaaui* strain PMMR-2 (NR_153719.1) from Acidobacteriaceae were identified and obtained from the NCBI Genbank database. The 16S rRNA sequences were aligned using ClustalW [44] in mega (v. X) [45]. A phylogenetic tree was constructed using maximum likelihood to estimate the relatedness of the aforementioned species. The maximum likelihood tree was constructed using the following settings: bootstrapping (100 replicates), substitution type (nucleotide), model (Tamura-Nei), ML heuristics (nearest-neighbor-interchange), initial tree (NJ/BioNJ).

### 2.6. RNA Extraction

RNA was extracted from triplicate *Acidiphilium* sp. C61 cultures grown to exponential phase (OD_600nm_ = 0.3) in APPW+YE medium with 0 or 10 µM PEA. Glucose (2 mM) was added to the medium to enhance growth. Biomass was harvested after 4 days and RNA was extracted using a modified version of the DNA extraction method described above. DNA was removed from 50 µL total nucleic acid extracts through enzymatic digestion with 2.5 µL (2 U µL^−1^) TURBO DNAse (Thermofisher Scientific, Waltham, MA, USA), 0.5 µL (20 U µL^−1^) RNase inhibitor and 5 µL 10X DNAase buffer (Thermofisher Scientific, Waltham, MA, USA) for 1 h at 37 °C. After digestion, successful DNA digestion was checked by agarose gel electrophoresis. Total RNA was purified using the RNA Clean & Concentrator-5 kit (Zymo Research, Freiburg, Germany), according to the manufacturer’s instructions. RNA purity was assessed by spectrophotometry, and RNA quantities were determined by fluorometry using Qubit RNA HS Assay (Life Technologies, Carlsbad, CA, USA) and a Qubit^®^ 3.0 fluorometer (Life Technologies, Carlsbad, CA, USA). RNA integrity was additionally assessed by agarose gel electrophoresis.

### 2.7. RNA-seq Library Preparation and Sequencing

Total RNA was subjected to library preparation using the NEBNext Ultra II directional RNA library prep kit for Illumina (New England Biolabs, Beverly, MA, USA), according to the manufacturer’s instructions. Size selection was used to select for fragments ranging between 150–200 bp in length. RNA libraries were quantified using a Qubit^®^ 3.0 fluorometer as described above and their fragment size range was assessed by high-resolution, chip-based gel electrophoresis with a Bioanalyzer 2100 instrument (Agilent Technologies, Waldbronn, Germany) and the Agilent DNA7500 Kit (Agilent Technologies, Waldbronn, Germany). Libraries were pooled equimolarly and sequenced in paired-end (2 × 150 bp) mode on a HiSeq 2000 instrument (Illumina, Munich, Germany). Transcriptome sequencing (RNA-seq) was performed by Eurofins Genomics (Constance, Germany).

### 2.8. RNA-seq Data Pre-Processing

Demultiplexing of raw data was done with bclfastq (v. 2.19) (Illumina). The quality of raw, demultiplexed RNA-seq datasets was assessed using FastQC (v. 0.11.7) (http://www.bioinformatics.babraham.ac.uk/projects/fastqc/). Raw data (raw reads) was adapter trimmed using trimgalore (v. 0.4.3, cutoff: 20) (https://www.bioinformatics.babraham.ac.uk/projects/trim_galore/) and filtered using sickle (v. 1.33, quality threshold: 20) (https://github.com/najoshi/sickle). Ribosomal RNA-derived sequences, as well as non-coding RNA sequences, were filtered out with SortMeRNA (v. 2.1) [46] using pre-compiled databases of SILVA [47] and Rfam [48]. The remaining, mRNA-derived sequences were mapped onto the assembled and annotated genome of *Acidiphilium* sp. C61 using bbmap (v. 38.12, settings: slow, k = 11) [49]. The resulting bam files were sorted and indexed with SAMtools (v. 1.3.1) [50]. Read count tables were generated from sorted and indexed bam files using featureCounts (v. 1.6.0) [51].

### 2.9. Differential Gene Expression Analysis

Differential gene expression analysis was carried out in the R framework for statistical computing (v. 3.5.1) (R Core Development Team, 2018) [52], using the package edgeR (v. 3.20.9), including all dependencies [53]. The analysis started from merged read count data of the two tested experimental conditions (10 μM PEA vs. 0 μM PEA supplementation). Pseudocounts, generated by log2-transforming counts+1 (where counts are equivalent to the raw counts per feature), were used for preliminary data exploration by generating M-A plots and multidimensional scaling. The biological coefficient of variation was calculated for each gene to assess biology-derived variation within replicate groups. Genes identified to be differentially expressed were false discovery rate (FDR) corrected and filtered with respect to log fold change, FDR-corrected *p*-value, and gene expression in counts per million (CPM).

### 2.10. Quantitative PCR

Total gene copy numbers of the *Acidiphilium* sp. C61 16S rRNA gene with and without PEA (0, 10, 50 µM; *n* = 3) were determined using quantitative PCR (qPCR), an Mx3000P instrument (Agilent Technologies, Waldbronn, Germany) and Maxima SYBR Green qPCR Mastermix (Agilent Technologies, Waldbronn, Germany). *Acidiphilium* sp. C61 16S rRNA gene copy numbers were determined using the primer set Bac8Fmod/Bac338Rabc [54,55] and previously published cycling conditions [56]. Genomic DNA was diluted to a range of 1–10 ng µL^−1^ and 0.6 to 6 ng of DNA was used as template. Standard curves were made using serial dilutions of plasmid-based standards carrying the amplicon defined by the used primer pair. These curves were linear for primer sets from 5 × 10^8^ to 5 × 10^1^ with R^2^ values of 0.999–1.000, and the qPCR performed with efficiencies between 80% and 90%. Welch’s *t*-test was used to compare treatments using the calculated quantification results and determine statistical significance (*p* < 0.05).

### 2.11. Quantification of eDNA Concentrations

eDNA concentrations were determined according to Tang et al. [57] in *Acidiphilium* sp. C61 incubations grown with increasing concentrations of PEA (0, 10, 50 µM; *n* = 3). Bacterial cells were removed from samples (approximately 900 µL) via centrifugation (4 min, 6800× *g*). The supernatant (700 µL) was transferred to a sterile 1.5 mL Eppendorf tube and mixed with 50 µL protein precipitation solution (Thermofisher Scientific, Waltham, MA, USA) by inverting (10 times) and centrifuged again (10 min, 12,100× *g*). Then, 700 µL of the supernatant was subsequently mixed with 70 µL 2.5 M NaCl and 1400 µL 96% ethanol prior to incubation at −20 °C for at least 24 h. DNA was precipitated by centrifugation (25 min, 4 °C, 18,300× *g*) and washed once in 70% (*v/v*) ice-cold EtOH. The pellet was air dried and resuspended in 50 µL TE buffer (10 mM Tris, 1 mM EDTA, pH 7.5) by vortexing for 25 s. The eDNA concentration was determined by fluorimetric quantitation.

### 2.12. Data Deposition

The *Acidiphilium* sp. C61 genome sequence has been deposited at the European Nucleotide Archive EBI-ENA under the Bioproject number PRJEB35789. The RNA-seq sequencing data has been deposited at the ArrayExpress under the accession E-MTAB-8619.

## 3. Results

### 3.1. Effect of PEA on Phenotype and Growth of Iron Snow Key Players

**Figure 1:**
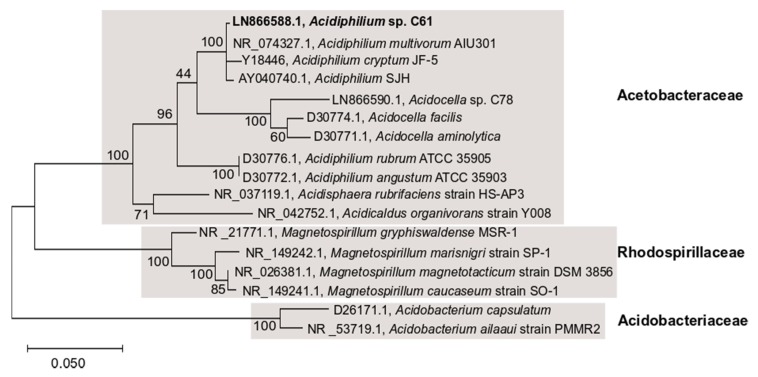
16S rRNA gene phylogenetic tree of *Acidiphilium* sp. C61 (bold) with other closely related isolates. The tree was reconstructed using the maximum likelihood method. GeneBank accession numbers for sequences are given. Scale bar shows 0.05 change per nucleotide position.

**Figure 2:**
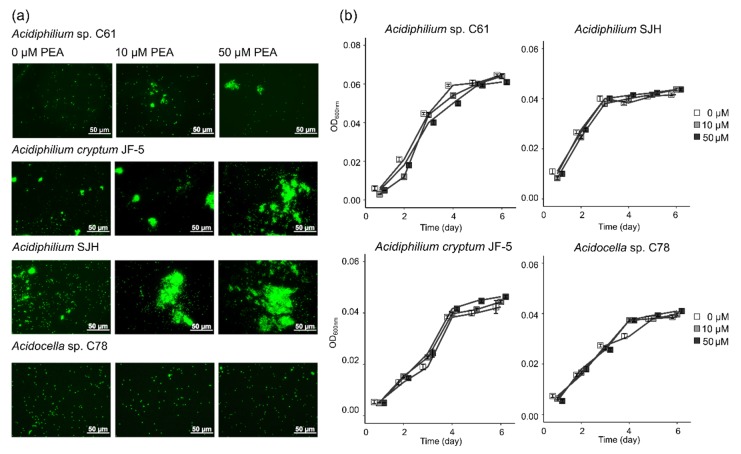
Effect of phenethylamine (PEA) on cell phenotype and growth of bacteria. (**a**) Fluorescence microscopy images of PEA-induced aggregate formation in *Acidiphilium* sp. C61, *Acidiphilium cryptum* JF-5, *Acidiphilium* SJH, and *Acidocella* sp. C78 incubations amended with increasing concentrations of PEA (0, 10, 50 µM) during aerobic growth. Total nucleic acids in subsamples taken after 3–4 days were stained with SYTO 13. (**b**) Aerobic growth curves (OD_600nm_) of *Acidiphilium* sp. C61, *Acidiphilium cryptum* JF-5, *Acidiphilium* SJH, and *Acidocella* sp. C78 (see methods for growth conditions) incubations grown with increasing PEA concentrations (0 µM, white square; 10 µM, grey square; and 50 µM, black square). Values represent means of triplicate samples (*n* = 3); error bars represent one standard deviation.

Given the previously observed, aggregation-inducing effect of PEA on *Acidiphilium* sp. C61 [17], we were interested in assessing the extent to which this phenotype is conserved across different *Acidiphilium* species and other iron reducers and oxidizers present in iron snow. Using three closely related *Acidiphilium* strains (Figure 1), we applied increasing concentrations of PEA (up to 50 μM) and used fluorescence microscopy to monitor any aggregate formation (Figure 2a). PEA induced aggregation in all tested *Acidiphilium* strains, with the greatest response observed in *Acidiphilium* SJH and *A. cryptum* JF-5, compared to *Acidiphilium* sp. C61. Higher concentrations of PEA resulted in increased numbers of aggregates formed (Figure 2a). In addition, biofilm formation of *Acidiphilium* sp. C61 on glass slides was enhanced in the presence of 10, 50, and 100 µM PEA after 5 days but not after 7 days.

For comparison, we also tested a potential response to PEA by the more distantly related *Acidocella* sp. C78 and the iron oxidizer *Ferrovum* sp. PN-J47, but we did not observe any aggregation (Figure 2a). Although PEA led to a distinct physiological response by *Acidiphilium* spp. following the addition of increasing concentrations of PEA, it did not have any effect on growth over time for the three tested *Acidiphilium* spp. or *Acidocella* sp. C78 (Figure 2b). However, rates of *Ferrovum* sp. PN-J47 iron oxidation increased 1.2- and 1.6-fold in the presence of 10 and 50 µM PEA, respectively (Figure S1).

### 3.2. Genome Sequencing, Assembly and Annotation of Acidiphilium sp. C61

We sequenced the genome of *Acidiphilium* sp. C61 to reconstruct its metabolic potential and to identify potential mechanisms enabling aggregate formation. Genome assembly led to a draft genome comprising six contigs (Table S1). The longest contig (326,6126 bp) accounted for 84.8% of the total assembly. Based on the presence and copy numbers of single-copy marker genes, the genome was 100% complete and showed a contamination of 2.24%. The assembled genome sequence of *Acidiphilium* sp. C61 has a size of 3.85 Mbp, a GC content of 66.1%, and contains 3700 open reading frames (ORF), of which 3604 are protein-coding genes and 96 are non-coding RNA genes (6 ribosomal RNA genes and 90 transfer RNA genes). Ninety transfer RNA genes comprised one tmRNA and multiple copies for all tRNA-genes except tRNA-Cys and tRNA-Trp. Among the 3604 protein-coding genes, 2155 genes were assigned a putative function and 1333 encode hypothetical proteins. In addition, we identified 116 transposase genes.

### 3.3. Potential Mechanisms of Aggregate Formation in Acidiphilium sp. C61

**Figure 3:**
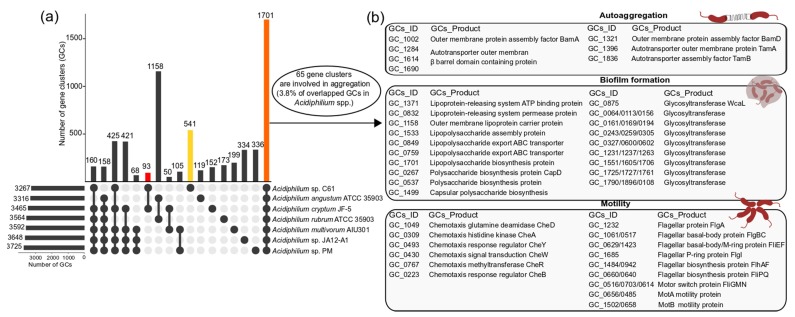
Genome comparison and identification of overlapping gene clusters (GCs) involved in bacterial aggregation of *Acidiphilium* sp. C61. (**a**) Intersect plot of the genome comparison of *Acidiphilium* sp. C61 with 6 other *Acidiphilium* sp. The horizontal bar chart in panel (a) corresponds to the total number of GCs found in each *Acidiphilium* strain. The vertical bar chart in panel (a) depicts the number of shared or unique GCs of intersected set under the corresponding species name and dark connected dots on the bottom panel indicate which *Acidiphilium* sp. is linked to each intersected set. The orange bar corresponds to the total number of overlapping GCs in all 7 *Acidiphilium* spp., the yellow bar corresponds to the total number of unique GCs found in *Acidiphilium* sp. C61, and the red bar corresponds to the total number of unique overlapping GCs between *Acidiphilium* sp. C61 and *Acidiphilium cryptum* JF-5, both of which were isolated from the same acidic lake (Lake77). Note, only overlapping GCs with a value greater than 50 are shown. (**b**) Schematic representation of a subset of overlapping GCs involved in aggregation. The GCs linked to these 3 categories (autoaggregation, biofilm formation, motility) were identified based on homologous functions to genes encoding for known aggregation mechanisms. Briefly, the 1701 shared GCs present in all *Acidiphilium* spp. were manually inspected based on annotated gene functions. Amino acid sequences of overlapping GCs linked to autoaggregation, biofilm formation, and motility were subset and compared to the *Acidiphilium* sp. C61 genome using BLAST (autoaggregation) or annotated using *dfast* (biofilm formation and motility). The IDs (GCs_ID) and corresponding annotated functions (GCs_Product) for the identified GCs are shown. In some cases, multiple GCs encode for the same function.

Given the previously observed phenotype of *Acidiphilium* sp. C61 forming aggregates upon exposure to PEA, our genome analysis focused on identifying mechanisms potentially involved in facilitating aggregate formation, including autoaggregation, motility, and biofilm formation. Since the aggregation effect was not specific to *Acidiphilium* sp. C61, we performed initial pangenome analyses of seven *Acidiphilium* spp. available in the GenBank Assembly database. The core genome of all *Acidiphilium* spp. analyzed included 1701 gene clusters, which comprised 52.1% of the gene clusters in the *Acidiphilium* sp. C61 genome (Figure 3a).

After screening the core genome for gene clusters relevant to aggregation, we observed that the seven strains shared 65 gene clusters (3.8% of overlapped gene clusters). These functions of these gene clusters are commonly associated with bacterial autoaggregation (autotransporter), motility (flagellar assembly, chemotaxis), and biofilm formation (exopolysaccharide biosynthesis and transport) (Figure 3b, Table S2-1) since the exact genes and mechanisms of three potential aggregations were not elucidated in previous studies. Out of the gene clusters, 541 were unique to *Acidiphilium* sp. C61, with the majority encoding hypothetical proteins (60%) (Table S2-2). Other strain-specific gene clusters encoded proteins involved in capsular polysaccharide biosynthesis, capsular polysaccharides transport (*kps*), the import of urea, and CRISPR/Cas systems, which function to protect against viral attack.

The inhibition of flagellar motility represents one mechanism of aggregation. Bacterial flagella consist of six components: basal body, motor, switch, hook, filament, and export apparatus, and screening the genome of *Acidiphilium* sp. C61 revealed genes for all six components (Table S3). Modulation of flagellar-based motility is facilitated in *Acidiphilium* sp. C61 by an intact chemotaxis pathway, including genes for methyl-accepting chemotaxis proteins (*mcp*) and two-component systems (*cheAW*, *cheY*) that transduce environmental signals and interact with the flagellar basal body (*fliGMN*) and motor proteins (*motAB*).

Next, we screened the genome for genes encoding autotransporters, which are outer membrane proteins that facilitate aggregation by binding to extracellular matrix components and the surface of other cells. One autotransporter gene and two genes coding for autotransporter modification proteins were identified in the *Acidiphilium* sp. C61 genome. In addition, we identified genes with a homology (amino acid sequence identity >30%) to other putative autotransporter genes (Table S3).

Extracellular polymeric substances (EPS) are considered to be one of the major structural components of the biofilm matrix that form on solid surfaces as well as non-surface attached aggregates, for example, pellicle biofilms that form at the air–liquid interface. Genes encoding proteins involved in the synthesis and secretion of exopolysaccharides, such as glycosyltransferases, the putative polysaccharide biosynthesis/export protein (*wza*), and the capsular polysaccharide export protein (*kps*) were identified. The presence of different pathways involved in exopolysaccharide precursor production suggests that EPS biosynthesis plays a role in *Acidiphilium* sp. C61 aggregate formation. Lastly, we looked for genes related to quorum sensing based biofilm formation and aggregation. Quorum sensing can induce aggregate formation through coordinated changes in gene expression mediated by autoinducers in response to cell density fluctuations, which can regulate biofilm formation. However, the *Acidiphilium* sp. C61 genome lacks autoinducer synthesis and receptor genes.

### 3.4. Central Metabolism and Iron Reduction Machinery in Acidiphilium sp. C61

**Figure 4:**
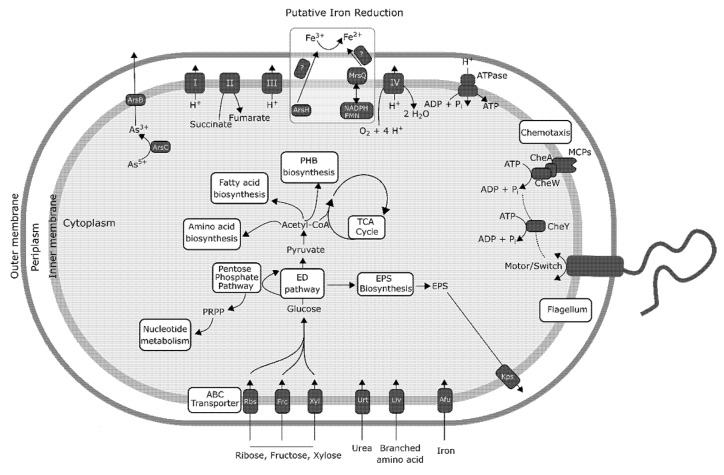
Genomic potential of *Acidiphilium* sp. C61, based on gene annotations described in the newly sequenced *Acidiphilium* sp. C61 genome. Here, the energy production pathway, oxidative phosphorylation pathway, sugar transporters, as well as all potential mechanisms (exopolysaccharide production, flagellar motility) of aggregate formation are included. Also shown are potential mechanisms of iron reduction derived from (1) previous publications [59,60] describing potential iron-reduction machinery in other acidic iron reducers, and (2) genes identified in the *Acidiphilium* sp. C61 genome with significant homology to iron reduction machinery described in other well-characterized iron-reducing bacteria, for example, *Shewanella*. ArsBC = arsenate reductase subunits B/C, MCPs = methyl-accepting chemotaxis proteins, CheA = chemotaxis protein CheA, CheY = chemotaxis protein CheY, EPS = extracellular polymeric substance, ED pathway = Entner–Doudoroff pathway, Rbs = ribose transport protein, Frc = fructose transport protein, Xyl = xylose transport protein, Urt = urea transport protein, Liv = branched-chain amino acid transport protein, Afu = iron transport protein, Kps = capsular polysaccharide export protein.

The lack of genomic information available for acidophilic FeRB prompted us to investigate the metabolic potential of *Acidiphilium* sp. C61 as a whole. The *Acidiphilium* sp. C61 genome encodes an incomplete glycolysis pathway but features full sets of genes required for the tricarboxylic acid (TCA) cycle and oxidative phosphorylation (Figure 4, Table S4). The lack of an intact glycolysis pathway is compensated by a complete pentose phosphate pathway. We found no genes related to major carbon fixation pathways, but *Acidiphilium* sp. C61 appears to be able to fix carbon dioxide heterotrophically since its genome encodes a pyruvate carboxylase (*pyc*) and a pyruvate orthophosphate dikinase (*ppdk*) similar to *Acidiphilium* sp. JA12-A1 [58]. *Acidiphilium* sp. C61 possesses genes encoding all pathways for the biosynthesis of proteinogenic amino acids, nucleotide and fatty acid biosynthesis (Figure 4). Multiple encoded transporters enable the transport and utilization of inorganic nutrients (e.g., *afuABC*) and organic substrates (e.g., *kpsET*). Among others, we identified genes encoding ribose (*rbsABC*), fructose (*frcABC*) and xylose (*xylFGH*) transporters. We also found seven genes of two complete pathways (starting from acetyl-CoA) responsible for the biosynthesis and accumulation of poly-β-hydroxybutyrate (PHB), a common storage material in prokaryotic cells typically synthesized in the presence of excess organic carbon and previously identified in *Acidiphilium cryptum* JF-5 [14,59] and *Acidiphilium* sp. JA12-A1 [58].

In comparison to neutrophilic and alkaliphilic FeRBs, our knowledge about the iron-reducing machinery in acidophiles is limited [60]. *Acidiphilium* sp. C61 possesses genes encoding for cytochrome c (Table S5-1), which is a outer-membrane cytochrome suggested to be involved in iron respiration of *A. cryptum* JF-5 [61]. No other conclusive iron reductases in acidophilic FeRB, such as *Acidocella* or *Acidiphilium* spp., have been identified to date [58,60]. We also identified a complete set of genes involved in oxidative phosphorylation and genes coding proteins known to be involved in electron transfer using a variety of electron donors such as NADH, NADPH, glutathione, and electron transfer mediators such as FMN and FAD (Table S5-2). These electron donors and electron transfer mediators are necessary to transfer electrons to Fe^3+^.

Furthermore, we searched for candidate iron reductase involved in iron reduction based on knowledge on other FeRB and electron transfer in general (Figure 4, Table S5-3). A homology search using all available iron reductases found in the NCBI non-redundant protein database against the genome of *Acidiphilium* sp. C61 was performed. Homology searches identified only one gene with a homology of 45.8% compared to *msrQ* (methionine sulfoxide reductase heme-binding subunit) from the neutrophilic FeRB *Shewanella* sp. Sh95 that could play a role in iron reduction. In addition, we identified a gene encoding an arsenate reductase (AcpC61_1183), which might function as iron reductase under acidic conditions [62].

### 3.5. Differential Gene Expression Analysis

We performed RNA-seq analysis on *Acidiphilium* sp. C61 with and without PEA exposure to examine transcriptome-wide responses to this aggregate-inducing chemical mediator. Under these specific growth conditions, we detected gene expression for 3598 out of 3604 genes encoded in the genome of *Acidiphilium* sp. C61. Gene expression ranged from 1.0 and 14.3 log_2_ counts per million (log_2_CPM). A detailed analysis of overall highly expressed genes (log_2_CPM >9) revealed primarily genes linked to carbohydrate metabolism, electron transfer, ATP synthesis, amino acid metabolism, and genetic information processing (DNA replication, transcription, protein biosynthesis) (Table S6). Except for a few seemingly random genes, genes linked to potential aggregation mechanisms were not among those featuring highest gene expression values. Gene expression for flagella biosynthesis, for instance, ranged between 6 and 10 log_2_CPM and between 5 and 9 log_2_CPM for chemotaxis.

**Figure 5:**
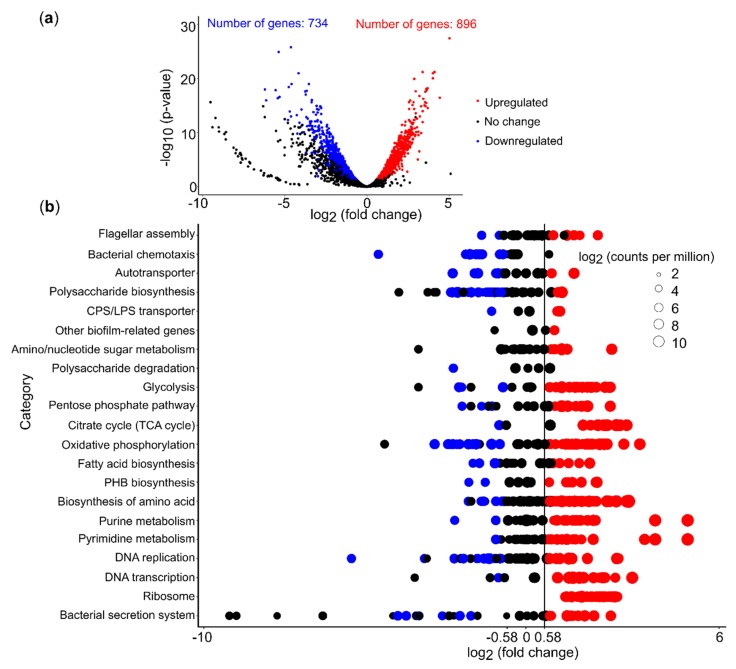
Differential gene expression profiling of *Acidiphilium* sp. C61 upon PEA (10 µM) supplementation. (**a**) Volcano plot of differentially expressed genes (DEGs) with statistical significance and fold change in the control and treatment group. Significantly differentially expressed genes are defined by a log_2_FC (log_2_ fold change) > 0.58 or log_2_FC < –0.58, log_2_ counts per million (log_2_ CPM) > 6, and an adjusted *p*-value FDR (false discovery rate) < 0.05. Each dot represents a single expressed gene. Red dots represent upregulated genes, blue dots represent downregulated genes, and black dots represent genes not upregulated or downregulated. (**b**) Gene expression patterns of selected KEGG pathways in *Acidiphilium* sp. C61 incubations with 10 μM PEA. Each bubble denotes one gene and the bubble size indicates log_2_ CPM values. Red and blue bubbles represent up- and downregulated genes, while black bubbles represent non-significantly differentially expressed.

PEA addition triggered a pronounced shift in gene expression. Out of 3598 expressed genes, 45.3% were differentially expressed in *Acidiphilium* sp. C61 plus PEA incubations (log_2_ fold change (FC) >0.58 or <–0.58, log_2_ counts per million (CPM) >6 and false discovery rate (FDR <0.05) (Figure 5a, Table S7). Additionally, 896 genes were upregulated and 734 genes were downregulated, which equates to 55% and 45%, respectively, of all differentially expressed genes. Among these differentially expressed genes, 254 (26.5% of upregulated genes) and 216 (28.6% of downregulated genes) genes encode hypothetical proteins (Table S7). We focused on genes linked to potential aggregation mechanisms (Figure 5b, Table S8). Out of 29 genes involved in flagella biosynthesis, 6 were upregulated, including *fliC* (coding for the flagella filament), *flgI* (flagella P-ring protein precursor), *flgB*, *flgC*, *flgG* (encoding flagella proximal and distal rod proteins), *fliL* (flagella basal body rod protein). Only two genes, *fliR* (flagellar biosynthetic protein), *flgA* (flagella basal body P-ring formation, AcpC61_0944) were downregulated and the other *flgA* gene copy (AcpC61_1269) remained unchanged (Figure 5b, Table S8). Chemotaxis related genes ranged in expression between 4 and 8 log_2_CPM. We observed that multiple *mcp* genes were downregulated, while other components of the chemotaxis machinery, for example, two-component systems, were not affected by PEA. For exopolysaccharide synthesis genes, the majority of genes involved in the exopolysaccharide precursor biosynthesis pathways (e.g., UDP-glucose, UDP-galactose) were upregulated, whereas genes involved in capsular polysaccharide biosynthesis were downregulated (e.g., glycosyltransferase, capsular polysaccharide biosynthesis protein) (Figure 5b, Table S8). Genes linked to capsular polysaccharide export (*kpsE*, *kpsT*) were upregulated.

### 3.6. PEA Induced Upregulation of Central Cellular Metabolism

**Figure 6:**
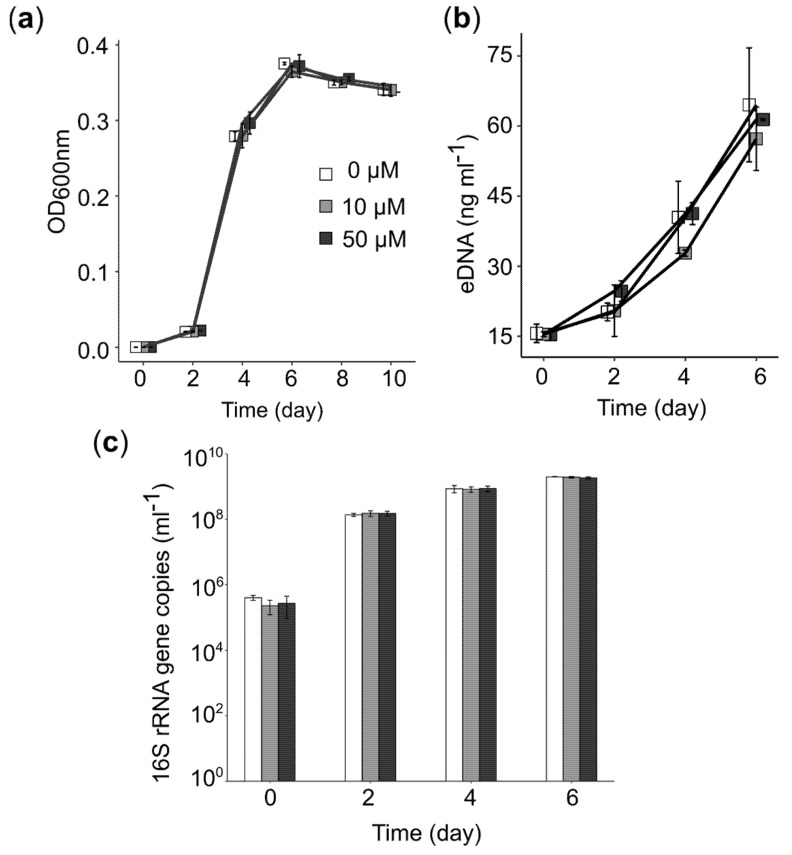
Effect of PEA on eDNA release and growth in *Acidiphilium* sp. C61. (**a**) Growth curve of *Acidiphilium* sp. C61 culture supplemented with different concentrations of PEA (0 µM, white; 10 µM, grey; 50 µM, black), *n* = 3. (**b**) eDNA concentration in cultures of *Acidiphilium* sp. C61 supplemented with different PEA concentrations. (**c**) Bacterial 16S rRNA gene copies numbers mL^−1^ on days 0, 2, 4, 6 of the incubation were quantified and compared among *Acidiphilium* sp. C61 cultures with and without PEA.

We also used RNA-seq data to assess the effect of PEA on genes linked to central cellular metabolism. More than 50% of the genes involved in glycolysis, the TCA cycle, and oxidative phosphorylation were upregulated (Figure 5b). Although we found that genes involved in energy production, for example, genes involved in DNA precursor biosynthesis, were highly expressed, we did not observe an increase in bacterial growth or production of eDNA (Figure 6). We did not detect a significant increase in bacterial 16S rRNA gene copies nor eDNA concentration in cultures of *Acidiphilium* sp. C61 in incubations supplemented with PEA despite enhanced aggregate formation. We also observed no change in the activity of DNA replication. However, we found that most genes involved in amino acid biosynthesis and transcription were upregulated (Figure 5b). Genes involved in the synthesis of ribosomes and genes encoding signal peptidases, which are involved in the removal of signal peptides from secretory proteins, were also upregulated. Genes linked to the Sec translocation pathway, which provides a major pathway of protein translocation from the cytosol across the cytoplasmic membrane in bacteria, were also upregulated (Table S8).

## 4. Discussion

Bacteria of the heterotrophic alphaproteobacterial genus *Acidiphilium* are ubiquitous in acidic environments [63]. These heterotrophs are often isolated as contaminants from iron-oxidizing mixed cultures composed of acidophiles like *Acidithiobacillus ferrooxidans* [64,65] or species related to *Ferrovum myxofaciens* P3G [58]. In these iron-oxidizing mixed cultures, *Acidiphilium* spp. enhance the activities of these chemolithoautotrophs in bioleaching. In return, *Acidiphilium* spp. seem to benefit from their secreted metabolites and biomass remnants [28,66]. *Acidiphilium* spp. have been also directly isolated from acidic mine drainage waters and sediments [14,67] and from acidic hypersaline river sediments in Australia, where they can make up high relative fractions of the microbial community [68].

Independent of their original ecological niche, all seven *Acidiphilium* spp. analysed by pangenomics show high similarities regarding their functional genome organization. Not surprisingly, both strains isolated from the same lake share the highest number of accessory gene clusters (93 gene clusters), with most of them being related to hypothetical proteins except a few related to transporters. Genes encoding different mechanisms of aggregation were present in all seven genomes, i.e., genes involved in the synthesis and secretion of EPS, suggesting that these mechanisms of aggregation are common in *Acidiphilium* spp. Indeed, all three *Acidiphilium* isolates tested in this study were able to aggregate to some extent, even without PEA addition. This morphological feature observed in *Acidiphilium* isolates has been previously documented, for example, the salt-tolerant *Acidiphilium* strain, AusYE3-1, also forms flocs and alters cell shapes from rod-shaped or coccobacillus to filamentous structures when stressed under high salt concentrations [68].

Our study shows PEA enhanced aggregation of all *Acidiphilium* strains tested, but not of other acidophiles [15,17] also present in iron snow. However, the PEA enhanced aggregate formation of *Acidiphilium* sp. C61 was less pronounced (Figure 2a) compared to the high number of large macroscopic cell aggregates formed by cultures of *Acidiphilium* sp. C61 soon after isolation from iron snow [17]. In that previous study, increased growth in the presence of 10 µM PEA was also observed, which could not be repeated in our study, suggesting adaptations during extended laboratory incubation of *Acidiphilium* sp. C61.

Based on our previous model [17], we anticipated that PEA induced gene expression changes would primarily be related to motility similar to its role in *Proteus mirabilis* [22,23]. However, the assembled genome of *Acidiphilium* sp. C61 lacks the *flhDC* gene cluster present in *P. mirabilis*, and flagellar motility was not negatively affected by PEA addition. Motility still seems to be essential for *Acidiphilium* sp. C61, as the six genes involved in flagella biosynthesis were even slightly upregulated. This finding agrees with the results of a metaproteomic approach, which detected many flagellin domain proteins from *Acidiphilium* spp. in iron snow samples [15]. Furthermore, chemotaxis sensor proteins were downregulated in the presence of PEA, enabling more smooth swimming. Thus, flagellar motility might help *Acidiphilium* sp. C61 join iron oxidizers, like *Acidithrix* sp. C25 in the growing aggregate, then again, there may not be sufficient time for the microorganism to switch from a pelagic to an attached lifestyle.

*Acidiphilium*, *Acidithrix*, *Acidocella,* and *Ferrovum* spp. can make up 53% of the total bacterial community of aggregates formed in acidic lignite lakes [15]. In these shallow lakes, iron snow forms a continuous shower of iron minerals, (in)organic matter and microorganisms (∼10^8^–10^10^ cells (g dry wt^−1^)) rapidly falling through the water column to the sediment [5,69,70]. Thus, there is only a short lifespan of these pelagic aggregates, which consequently means there is only limited time for microbial-coordinated activities, and for energy and matter fluxes to occur within these aggregates. Although acyl-homoserine lactone (AHL) mediated gene regulation has been shown to influence EPS production and biofilm formation in many proteobacteria, including *A. ferrooxidans* [71], we could not find autoinducer synthesis or receptor genes linked to quorum sensing in the genome of *Acidiphilium* sp. C61. Thus, communication appears to occur via other interaction mechanisms mediated by diffusive exometabolites (infochemicals).

Bacterial EPS is usually composed of a mixture of polysaccharides, proteins, lipids, and extracellular DNA (eDNA) [72,73]; however, the main constituents of EPS extracted from *Acidiphilium* strain 3.2Sup(5) are proteins and carbohydrates mostly composed of carboxylic, hydroxylic, and amino groups [74]. Although we observed the upregulation of several genes for exopolysaccharide precursor synthesis (e.g., UDP-glucose, UDP-galactose) and capsular polysaccharide exporters in the presence of PEA, the overall expression pattern of genes involved in polysaccharide synthesis, as well as autotransporters, were inconsistent. Thus, we cannot conclude that biofilm formation, in general, is enhanced in the presence of PEA, nor can we explicitly conclude the mechanisms involved in *Acidiphilium* sp. C61 biofilm formation. Similarly, we did not detect significantly enhanced eDNA concentrations, indicating eDNA is likely not a primary constituent of EPS secreted by *Acidiphilium* sp. C61 and *Acidiphilium* sp. C61 may prefer to aggregate with other cells over forming biofilms.

The high surface area of the poorly crystalline iron mineral schwertmannite, which forms the inorganic matrix of iron snow [13,69], favors adsorption of organic matter that are ideal substrates for *Acidiphilium* spp. [14,15]. The above mentioned metaproteomic approach also identified *Acidiphilium*-related glucose uptake proteins in iron snow [15]. The genome of *Acidiphilium* sp. C61 contains ABC transporters for the uptake of ribose, fructose, and xylose (Figure 4). In contrast to the genome of *Acidiphilium* sp. JA12-A1 that lives in co-culture with *Ferrovum* sp. JA12 [58], we did not find polysaccharide-hydrolyzing enzymes, such as β-glucosidases, or break down EPS or cell envelope polysaccharides from decaying cells endoglucanases in *Acidiphilium* sp. C61. However, glycoside hydrolase, alpha-amylase, beta-N-acetylhexosaminidase, and glucoamylase were present in all *Acidiphilium* spp. based on the pangenomic analysis (GC_1878, GC_1672, GC_1296, GC_1827) (Table S2-3). In addition, *Acidphilium* sp. C61 possesses one more unique glycoside hydrolase (GC_6119), whereas another glycosidase (GC_1572) is present in the other six *Acidiphilium* strains. The capacity for polysaccharide degradation seems to be a common trait for *Acidiphilium* spp., but individual differences exist between the strains. Thus, these individual differences allow for niche differentiation and also ensures complementarity, since a diverse mixture of strains will colonize specific habitats.

In general, sugar compounds appear to be the preferred carbon source for biomass production in all *Acidiphilium* sp. We identified full sets of genes of the pentose phosphate pathway, compensating for the incomplete glycolysis pathway, a complete tricarboxylic acid (TCA) cycle, and genes encoding all pathways necessary for the synthesis of proteinogenic amino acids, nucleotide, and fatty acid biosynthesis. *Acidiphilium* sp. C61 is capable of urea uptake, a unique trait among *Acidiphilium* sp. Thus, it can be characterized as a prototrophic cell, able to synthesize all the compounds needed for growth listed above without the need for a partner organism. Different *Acidiphilium* strains present in complex communities appears to release a diverse suite of glycoside hydrolases and glucosidases to utilize the organic substances secreted by other community members or derived from microbial cell decay. In return, *Acidiphilium* spp. provide the chemolithoautotrophs with elevated CO_2_ concentrations locally, which is advantageous especially in low pH environments, such as acidic coal mining lakes. This type of interspecies carbon transfer has been previously described for acidophilic mixed cultures containing *Acidiphilium cryptum* and *Acidithiobacillus ferrooxidans* [75] and other mixed cultures derived from a pilot plant for remediation of acid mine drainage (AMD) containing *Acidiphilium* sp. JA12-A1 and an iron oxidizer related to *Ferrovum myxofaciens* P3G [76].

To our surprise, PEA did not preferentially affect one or more mechanisms of aggregate formation in *Acidiphilium* but induced upregulation of the central cellular metabolism by affecting more than 50% of the genes involved in glycolysis, the TCA cycle, and oxidative phosphorylation. Similarly, the synthesis of ribosomes, amino acid biosynthesis and transcription, as well as secretion systems, were stimulated. This broad range of affected upregulated genes points to a more general stimulatory mechanism of PEA, similar to its general role as a neurotransmitter [18] and stimulator for the release of biogenic amines in humans [20]. Thus, it is probable that these *Acidiphilium* cells are just more active in iron snow in the presence of the infochemical PEA released by *Acidithrix* sp. C25.

After the formation of iron minerals at the oxic-anoxic interface, iron snow will reach anoxic conditions in the hypolimnion. Since Fe^3+^ is energetically much more favorable as an electron acceptor at acidic compared to pH neutral conditions [77], the majority of the chemolithoautotrophic Fe^2+^ oxidizers are also capable of Fe^3+^ reduction, including *Acidithrix* sp. C25 [13]. These heterotrophic *Acidiphilium* spp., as well as other heterotrophic acidophiles, are also capable of Fe^3+^ reduction even in the presence of oxygen [78,79,80]. Thus, single cells within the iron snow aggregates may begin to respire Fe^3+^ in the redoxcline, even at low oxygen concentrations. Switching to this anaerobic metabolism requires activation, as genes responsible for Fe^3+^ reduction in *Acidiphilium* spp. do not seem to be constitutively expressed [78]. Although the Fe^3+^ reduction mechanism in *Acidiphilium* spp. has not yet been revealed in detail, different membrane-associated proteins potentially related to electron transport chain genes have been identified in iron snow, including OmpA/MotB domain proteins, TonB-dependent receptor, and ApcA [15]. Genome assembly of *Acidiphilium* sp. C61 reveals MsrQ that can bind to two b-type hemes via conserved histidine residues along with MsrP; these proteins form a methionine sulfoxide reductase operon functioning to repair oxidized periplasmic proteins [81]. Additionally, the cytosolic NAD(P)H flavin reductase (Fre) has been shown to function as a proficient electron donor to MsrQ moieties and the soluble dehydrogenase partner, in *Escherichia coli*, for example [81]. These findings suggest that Fre and MsrPQ might form a membrane-spanning two-component system for electron transfer (Figure 4). Because MsrPQ is involved in oxidative stress response, specifically in the repair of oxidized periplasmic proteins, such as oxidized methionine residues, there is a potential role for the MsrPQ operon in the maintenance of the activated methyl cycle, which can be remotely linked to iron reduction via the transsulfuration pathway. We also identified a gene coding for an arsenate reductase (AcpC61_1183). Previous studies suggest that TetH or ArsH have the potential to mediate Fe^3+^ reduction in acidophiles [62,82,83]. However, since we did not perform RNA-seq analysis of *Acidiphilium* sp. C61 under iron-reducing conditions, we do not know how PEA would affect its anaerobic metabolism.

## 5. Conclusions

Aggregation appears to be a common mechanism in all *Acidiphilium* spp., since nearly 4% of their shared gene clusters are associated with mechanisms responsible for aggregation, including autoaggregation, motility (flagellar assembly, chemotaxis), and biofilm formation (exopolysaccharide biosynthesis and secretion). All genes associated with these mechanisms were transcribed under our incubation conditions; however, RNA-seq data did not show clear evidence that PEA affected aggregate formation directly. Inconsistent gene expression patterns relating to the formation and secretion of EPS and flagellar-based motility, despite enhanced aggregate formation with the addition of PEA, suggests this compound functions as an infochemical regulating other cellular mechanisms, and not aggregation mechanisms directly. In fact, *Acidiphilium* cells seem to retain motility within the aggregates. We did observe induced upregulation of glycolysis, the TCA cycle, oxidative phosphorylation, and synthesis of ribosomes, although these activities were not linked to enhanced growth. Degradation of polysaccharides appears to be a major function within the heterotrophic Alphaproteobacterial genus *Acidiphilium*, which is optimized by the complementarity of specific genes present in unique strains in addition to shared core functions.

## Supplementary Materials

Supplementary materials can be found at https://www.mdpi.com/2076-2607/8/3/314/s1. Figure S1: Fe(II) consumption of *Ferrovum* sp. PN-J47 supplemented with different concentrations of PEA (0, 10 and 50 µM), Table S1: Genome features of *Acidiphilium* sp. C61 in comparison to six other *Acidiphilium* spp., Table S2: Gene clusters (GCs) involved in aggregation, unique gene clusters, and polysaccharide degradtion in *Acidiphilium* sp. C61 by Pangenomics, Table S3: Aggregation genes identified in the genome of *Acidiphilium* sp. C61, Table S4: Genes of central metabolism present in the genome of *Acidiphilium* sp. C61, Table S5: Iron-reduction related genes (cytochrome c, electron transfer, iron reductase) identified in *Acidiphilium* sp. C61, Table S6: Top 10% of highly expressed genes of *Acidiphilium* sp. C61 in the presence of 10 µM PEA, Table S7: Summary of differential expressed genes in *Acidiphilium* sp. C61 after 10 µM PEA addition, Table S8: General gene expression pattern in *Acidiphilium* sp. C61 supplemented with 10 µM PEA addition.
