# Supplementary figures and images for: Molecular Mechanisms Underpinning Aggregation in Acidiphilium sp. C61 Isolated from Iron-Rich Pelagic Aggregates
Source: Microorganisms. 2020 Feb 25;8(3):314. doi: 10.3390/microorganisms8030314 (PMC7142476; doi:10.3390/microorganisms8030314)

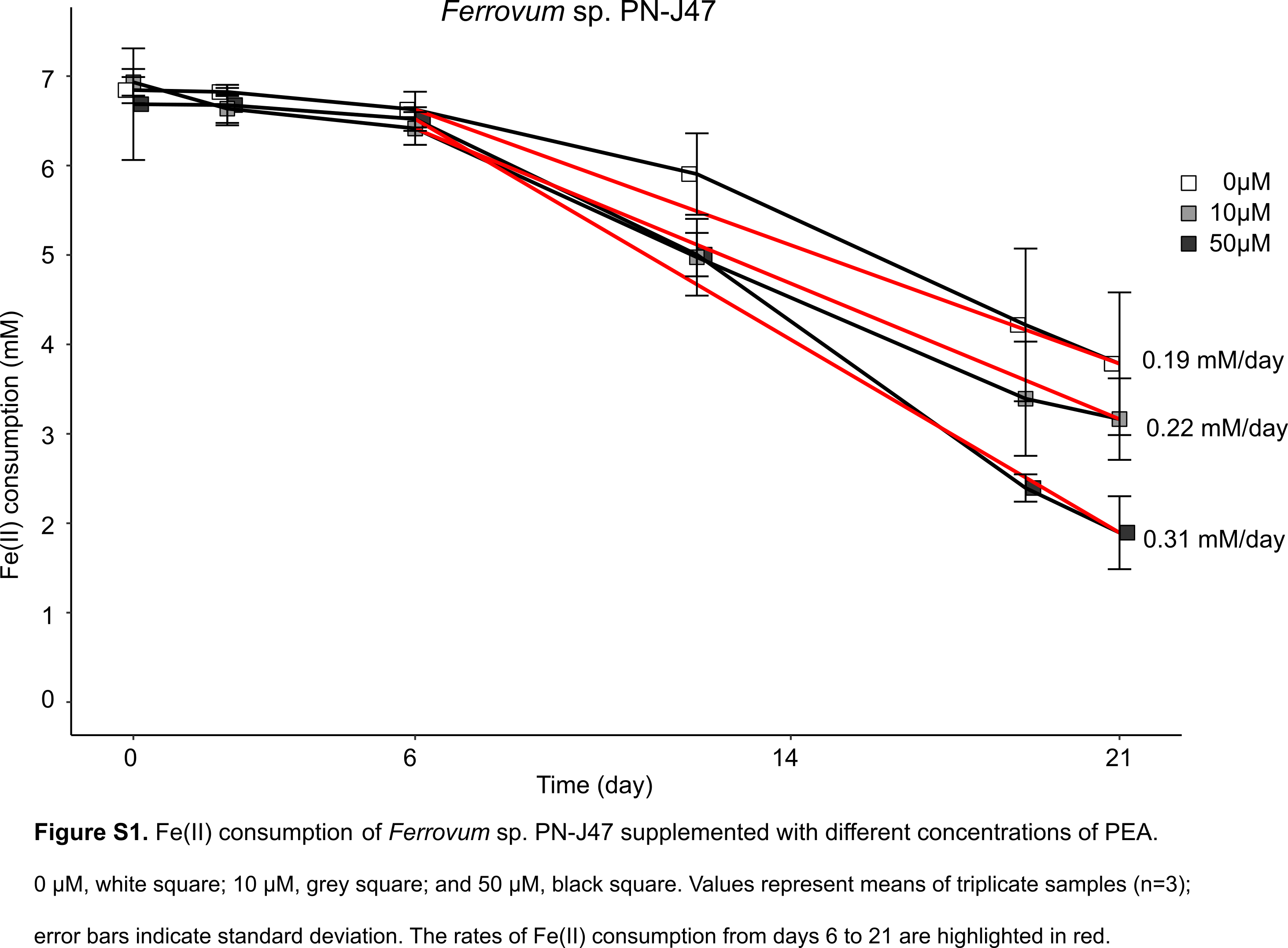

Supplement: Supplementary file 1 [file microorganisms-08-00314-s001.zip › Figure S1.png]
